# A possible cause of epistaxis: increased masked hypertension prevalence in patients with epistaxis^☆^

**DOI:** 10.1016/j.bjorl.2016.01.007

**Published:** 2016-04-18

**Authors:** Baran Acar, Bunyamin Yavuz, Erdem Yıldız, Selcuk Ozkan, Mehmet Ayturk, Omer Sen, Onur Sinan Deveci

**Affiliations:** a Kecioren Training and Research Hospital, Department of Otorhinolaryngology, Ankara, Turkey; b Medical Park Ankara Hospital, Department of Cardiology, Yenimahalle, Turkey; c Kecioren Training and Research Hospital, Department of Cardiology, Ankara, Turkey

**Keywords:** Masked hypertension, Epistaxis, Spontaneous, Hipertensão mascarada, Epistaxe, Espontânea

## Abstract

**Introduction:**

Epistaxis and hypertension are frequent conditions in the adult population. Masked hypertension is defined as a clinical condition in which a patient's office blood pressure level is <140/90 mmHg, but the ambulatory or home blood pressure readings are in the hypertensive range. Many studies have proved that hypertension is one of the most important causes of epistaxis. The prevalence of this condition in patients with epistaxis is not well defined.

**Objective:**

This study aimed to evaluate the prevalence of masked hypertension using the results of office blood pressure measurement compared with the results of ambulatory blood pressure monitoring.

**Methods:**

Sixty patients with epistaxis and 60 control subjects were enrolled in the study. All patients with epistaxis and controls without history of hypertension underwent physical examination, including office blood pressure measurement, ambulatory or home blood pressure, and measurement of anthropometric parameters.

**Results:**

Mean age was similar between the epistaxis group and the controls – 21–68 years (mean 42.9) for the epistaxis group and 18–71 years (mean 42.2) for the control group. A total of 20 patients (33.3%) in the epistaxis group and 7 patients (11.7%) in the control group (*p* = 0.004) had masked hypertension. Night-time systolic blood pressure was significantly higher in patients with epistaxis than in the control group (*p* < 0.005). However, no significant difference was found in daytime systolic blood pressure between the control group and the patients with epistaxis (*p* = 0.517).

**Conclusion:**

This study demonstrates increased masked hypertension prevalence in patients with epistaxis. We suggest that all patients with epistaxis should undergo ambulatory or home blood pressure to detect masked hypertension, which could be a possible cause of epistaxis.

## Resumo

**Introdução:**

Epistaxe e hipertensão são condições frequentes na população adulta. Hipertensão mascarada é definida como uma condição clínica em que o nível da pressão arterial do paciente no consultório é < 140/90 mm Hg, mas as leituras da pressão arterial ambulatorial ou em casa, se encontram na faixa hipertensiva. Muitos estudos demonstraram que a hipertensão é uma das causas mais importantes de epistaxe. Ainda não está devidamente definida a prevalência dessa condição em pacientes com epistaxe.

**Objetivo:**

Esse estudo teve por objetivo avaliar a prevalência de HM com o uso dos resultados de mensurações da pressão arterial no consultório, em comparação com os resultados da MAPA.

**Método:**

Para o estudo, foram recrutados 60 pacientes com epistaxe e 60 indivíduos para controle. Todos os pacientes com epistaxe e os controles sem histórico de hipertensão passaram por exame físico, inclusive determinação da pressão arterial no consultório, MAPA e mensuração dos parâmetros antropométricos.

**Resultados:**

A média de idade foi similar entre o grupo com epistaxe e os controles: de 21 a 68 (média 42,9) anos para o grupo com epistaxe, e de 18 a 71 (média 42,2) anos para o grupo controle. No total, 20 pacientes (33,3%) no grupo com epistaxe e 7 pacientes (11,7%) no grupo controle (*p* = 0,004) apresentaram hipertensão mascarada. A pressão arterial sistólica noturna foi significantemente mais alta em pacientes com epistaxe, em comparação com o grupo controle (*p* < 0,005). No entanto, não foi observada diferença significante na pressão arterial sistólica obtida durante o dia entre o grupo controle e os pacientes com epistaxe (*p* = 0,517).

**Conclusão:**

O presente estudo demonstra maior prevalência de hipertensão mascarada em pacientes com epistaxe. Sugerimos que todos os pacientes com epistaxe devam ser submetidos à monitoração da pressão arterial caseira ou em consultório com o objetivo de detectar hipertensão mascarada, que pode ser uma causa possível de epistaxe.

## Introduction

Bleeding in the nasal cavity caused by mucosa injury or vascular pathology as a result of coagulation disorders is known as epistaxis.^1^ Epistaxis is one of the most common ear–nose–throat (ENT) emergencies that require hospital admission, but is rarely life threatening. Epistaxis is more common in men than in women, and its frequency increases with age.^1^, ^2^ The incidence of epistaxis is not exactly known, but it is approximately 7–60% of the population. Epistaxis can be post-traumatic, iatrogenic (nasal surgery or endoscopic procedures), and spontaneous, resulting from possible causative factors, including local nasal factors (inflammation and infection), medications, and systemic factors such as coagulation disorders, alcoholism, hereditary hemorrhagic telangiectasia, and hypertension.^1^, ^2^

The nose has a rich vascular supply, with substantial contributions from the internal carotid artery (ICA) and the external carotid artery (ECA). The ECA system supplies blood to the nose via the facial and internal maxillary arteries. The ICA contributes to nasal vascularity through the ophthalmic artery.^3^ Kiesselbach's plexus or Little's area is an anastomotic network of vessels located in the anterior cartilaginous septum. Many of the arteries supplying the septum have anastomotic connections at this site. More than 90% of bleedings occur in the anterior region and arise from Little's area, where Kiesselbach's plexus forms on the septum.^3^ The posterior epistaxis, which is usually more profuse and arterial in origin, occurs further back in the nasal cavity. It has a greater risk of airway compromise, aspiration of blood, and greater difficulty in controlling bleeding.

Hypertension is a major cause of spontaneous epistaxis. Patients with epistaxis commonly present with elevated blood pressure. Epistaxis is more common in hypertensive patients, perhaps owing to vascular fragility from the long-standing disease.^4^ However, epistaxis patients with normal blood pressure are not well investigated for masked hypertension (MH).

The phenomenon of MH is defined as a clinical condition in which a patient's office blood pressure (BP) level is <140/90 mmHg, but the ambulatory or home BP readings are in the hypertensive range.^4^ The high prevalence of MH suggests the necessity for measuring out-of-office BP in persons with apparently normal or well-controlled office BP.^5^ The prevalence of MH in the general population could be as high as 10%. However, data obtained from several cross-sectional studies have demonstrated large differences, with prevalence rates ranging from a low of 8% to a high of 49%.^6^, ^7^ Hypertension by 24 h ambulatory blood pressure monitoring (ABPM) is defined when the mean daytime systolic BP is equal to or greater than 135 mmHg or when the mean daytime diastolic BP is equal to or greater than 85 mmHg, according to the seventh report of the 2003 US Hypertension Joint National Committee and European Society of Hypertension.^8^

This study aimed to evaluate the prevalence of MH using ABPM among patients with epistaxis.

## Methods

This prospective study included the review of the medical charts of patients with mild, moderate, or severe epistaxis that was treated medically or surgically between December 2012 and January 2015. All subjects gave their informed consent. The study protocol has been approved by the ethics committee of the Kecioren Hospital (approval No. 185-09.01.2013).

A total of 120 patients participated in the study. Patients were separated into two groups: the epistaxis group and the control group that did not present with epistaxis. Both groups included 60 patients, and each group had 40 male and 20 female patients. Patients’ age ranged from 21 to 68 years (mean 42.9) for the epistaxis group and 18 to 71 years (mean 42.2) for the control group. The inclusion criterion was spontaneous epistaxis (without trauma or nasal surgery) with normal office BP and without known hypertension. The exclusion criteria included chronic liver disease, chronic kidney disease, or coagulopathy, as well as patients who were taking anti-thrombotic drugs, had nasal trauma, had undergone nasal surgery, or had previous hypertension.

After patients’ BP was measured and active nose bleeding was stopped by medical or surgical management, laboratory parameters of the CBC, INR, and APTT tests were studied, echocardiography in the cardiology clinic was conducted, and a 24 h BPM device (Holter device) was applied to each patient.

Normal office BP was defined as <140/90 mmHg. MH was defined as a patient's office BP level of <140/90 mmHg and ABPM parameters in the hypertensive range (24 h average BP ≥ 130/80 mmHg and/or daytime average ≥135/85 mmHg and/or night-time average ≥120/70 mmHg).

### Statistical analysis

Continuous parametric and nonparametric data are presented as the mean ± standard deviation (SD) or as the median (range), respectively. Non-continuous variables are presented as percentages. Categorical variables were compared with Pearson's chi-squared test. Continuous variables were compared with Student's *t*-test and Mann–Whitney *U* tests. SPSS v. 15.0 was used for statistical analyses.

## Results

A total of 60 patients with epistaxis and 60 control subjects were enrolled in the study. The mean age was similar between the epistaxis group and the controls: 21–68 years (mean 42.9) for the epistaxis group and 18–71 years (mean 42.2) for the control group. No significant difference was found in the demographic features and laboratory and transthoracic echocardiography parameters between the groups.

**Figure 1:**
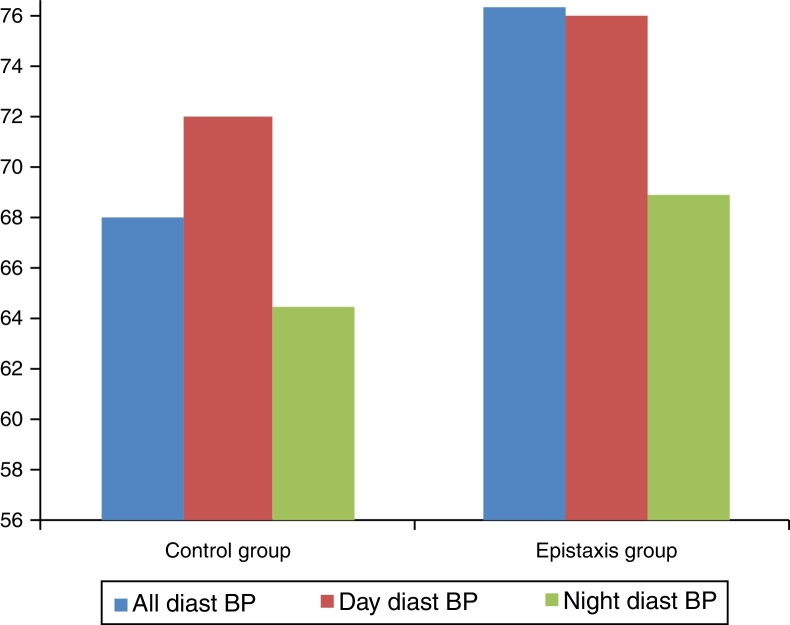
Day and night diastolic blood pressures (BP) were significantly higher for the epistaxis group.

**Figure 2:**
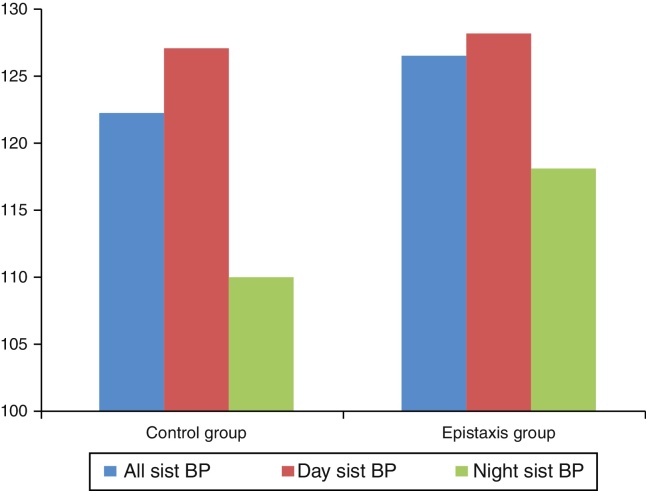
Night systolic blood pressure (BP) difference was found to be higher between the control and epistaxis groups.

Twenty patients (33.3%) in the epistaxis group and seven patients (11.7%) in the control group (*p* = 0.004) had MH. Figure 1, Figure 2 show the systolic and diastolic BP levels in the epistaxis and control groups. The office BP and ABPM parameters are presented in Table 1.

**Table 1:** Office blood pressure, 24 h ambulatory blood pressure, and their comparison for the control and epistaxis groups.

	Control (*n* = 60)	Epistaxis (*n* = 60)	*p*
	Mean, range	Mean, range	
*Office blood pressure (mmHg)*			
Systolic	120.3 (128–110)	120.1 (128–110)	0.817
Diastolic	74.2 (85–58)	74.0 (85–55)	0.840

*Average ABPM BP (mmHg) 24H*			
Systolic	121.1 (134–114)	125.6 (149–99.5)	0.012
Diastolic	67.2 (87–51)	75.7 (116–55)	0.00

*Day*			
Systolic	126.1 (145–107)	127.2(149–91)	0.517
Diastolic	71.4 (90–55)	75.1 (95–54)	0.009

*Night*			
Systolic	109.2 (120–99)	117.2 (139–88)	0.00
Diastolic	63.8 (75–48)	68.2 (88–48)	0.003

Daytime diastolic BP, 24 h systolic and diastolic BP, and nighttime systolic and diastolic BP were significantly higher in patients with epistaxis than in those in the control group. However, no significant difference was found in the daytime systolic BP between the control group and the patients with epistaxis (*p* = 0.517).

## Discussion

To the best of the authors’ knowledge, this study is the first to investigate MH prevalence in patients with epistaxis. The study shows increased MH prevalence in patients with epistaxis.

Epistaxis and hypertension are frequent conditions in the adult population. The relationship between the level of arterial pressure and the incidence of epistaxis in patients with hypertension is an issue that appears frequently in clinical practice.

Despite overt hypertension representing the major cause of spontaneous epistaxis, no study has yet evaluated the relationship between MH and epistaxis. Compared with other measures, the 24 h ABPM is more valuable for predicting prognosis, as it more accurately assesses the risk of cardiovascular disease than measurements of BP made during clinic or office visits, and ABPM is also closely related to damage of the target organ.^9^

Only a limited number of ABPM studies have examined patients with epistaxis. The probable reason is that if a patient presents with normal office BP, hardly any otolaryngologist would perform ABPM.^10^ Recent studies on the management of patients with epistaxis and hypertension do not mention the prevalence of MH in epistaxis patients, and only recommend the use of ABPM or home monitoring devices for a more accurate diagnostic of MH.^11^

For the purpose of this study, patients with epistaxis with no history of hypertension were deliberately chosen. This study found a high prevalence of MH in patients with epistaxis. MH was present in 20 (33.3%) patients in the epistaxis group and in seven (11.7%) patients in the control group. Page et al. showed that serious spontaneous epistaxis could also be the presenting sign of an underlying overt hypertension in about 43% of patients with no history of hypertension.^12^ This finding could be attributed to the selection of patients, as Page et al. included patients with serious epistaxis only. Comparing the present results with those in the available literature, it can be hypothesized that MH is more prevalent in patients with epistaxis than in those without. Based on these results, more large-scale studies that include subjects from the whole spectrum of patients with epistaxis and arterial hypertension are needed.

The present study used more common but stricter criteria for MH. The criteria are justified for use in patients with epistaxis because of the high prevalence of MH in these patients. Moreover, the nighttime systolic BP was significantly higher in patients with epistaxis than in those without. This finding may present evidence on the greater importance of nocturnal hypertension in the pathophysiology of the development of MH than the simple presence of daytime BP. More studies are needed for a better understanding of the pathophysiology of MH development.

The mechanism of how MH could lead to epistaxis remains unknown. One of the mechanisms may be related to endothelial dysfunction. A study revealed that the presence of MH is one of the independent determinants of cardiovascular disease.^6^ Only a limited number of studies have been conducted on how epistaxis influences MH or nocturnal hypertension. Proper blood pressure management is necessary for the prevention of persistent epistaxis from Kiesselbach's area in the clinical setting of emergency care practice.^13^ In the authors’ clinical practice, most epistaxis patients were admitted to the hospital at night, and MH was effective in showing cardiovascular organ damage. Therefore, ABPM should be performed in these patients.

## Conclusion

This study demonstrated that MH prevalence is higher in patients with epistaxis. It is suggested that all patients with epistaxis should undergo ABPM to detect MH, which could be a possible cause of epistaxis.

## Conflicts of interest

The authors declare no conflicts of interest.
